# Charting the way forward for HTA in Asia-Pacific: HTAsiaLink’s strategic plan

**DOI:** 10.1017/S0266462325100573

**Published:** 2025-12-19

**Authors:** Ryan Jonathan Sitanggang, Lapad Pongcharoenyong, Natcha Kongkam, Wendy Babidge, Auliya Suwantika, Izzuna Mudla Mohamed Ghazali, Ling-Chen Chien, Miyoung Choi, Saudamini Vishwanath Dabak, Sitanshu Kar, Takashi Fukuda, Wanrudee Isaranuwatchai, Yot Teerawattananon, Benjamin Shao Kiat Ong

**Affiliations:** 1 Health Intervention and Technology Assessment Program Foundation (HITAP), Nonthaburi, Thailand; 2 The Australian Safety and Efficacy Register of New Interventional Procedures – Surgical (ASERNIP-S), Adelaide, SA, Australia; 3 Indonesia Health Technology Assessment Committee (InaHTAC), Ministry of Health of the Republic of Indonesia, Jakarta, Indonesia; 4 Department of Pharmacology and Clinical Pharmacy, Faculty of Pharmacy, Universitas Padjadjaran (UNPAD), Bandung, Indonesia; 5 Malaysian Health Technology Assessment Section (MaHTAS), Ministry of Health Malaysia, Putrajaya, Malaysia; 6 Division of Health Technology Assessment (HTA), Center for Drug Evaluation (CDE), Taipei, Taiwan; 7 National Evidence-Based Healthcare Collaborating Agency (NECA), Seoul, South Korea; 8 Department of Social and Preventive Medicine, Jawaharlal Institute of Postgraduate Medical Education and Research (JIPMER), Puducherry, India; 9 Center for Outcomes Research and Economic Evaluation for Health (C2H), National Institute of Public Health, Government of Japan Ministry of Health Labour and Welfare, Saitama, Japan; 10 Institute of Health Policy Management and Evaluation, University of Toronto, Toronto, ON, Canada; 11 Saw Swee Hock School of Public Health, National University of Singapore (NUS), Singapore; 12 Agency for Care Effectiveness (ACE), Government of Singapore Ministry of Health, Singapore

**Keywords:** Health Technology Assessment, HTAsiaLink, strategic plan, network, Asia-Pacific, evidence-informed decision-making, capacity building

## Abstract

Health Technology Assessment (HTA) informs resource allocation and policy decisions, particularly to achieve Universal Health Coverage (UHC). Recognizing the increasing demand for evidence-informed decision-making, the HTAsiaLink network was established in 2011 as a regional platform to strengthen individual and institutional capacity in HTA research and facilitate the integration of HTA evidence into policy decisions across the Asia-Pacific.

Over the years, HTAsiaLink has expanded to over fifty members from twenty economies. In 2024, a structured strategic planning process was undertaken to ensure its continued growth and strengthen its impact on HTA development and implementation. This process involved a targeted review of strategic plans from international networks, alongside comprehensive member engagement, to develop a data-driven and adaptable plan responsive to the evolving healthcare landscape and member needs. As a result, five strategic priorities, corresponding action items, and success indicators were identified.

This commentary outlines the needs and processes involved in developing the network’s first-ever strategic plan, emphasizing the critical role of member engagement in shaping its future direction. We believe that this experience offers transferable insights for other HTA networks, particularly those operating in low- and middle-income country contexts, on the collaborative development of strategic plans that are responsive to shared objectives, accommodate varying institutional capacities, and align with regional priorities.

## Introduction

Health Technology Assessment (HTA) plays a critical role in informing resource allocation and policy decisions, particularly in achieving Universal Health Coverage (UHC) (1). In response to the increasing demand for HTA in Asia, the HTAsiaLink network was established in 2011 as a regional platform to strengthen HTA capacity, enhance knowledge exchange, and reduce duplication of efforts (2). The network functions on a voluntary basis, promoting collaboration among HTA agencies without the requirement of membership fees, and was established without seed funding, relying entirely on the voluntary engagement and strong commitment of its members. HTAsiaLink fosters collaboration through knowledge-sharing initiatives, capacity-building programs, and joint research projects, with the annual HTAsiaLink Conference serving as its flagship event. Over the past 14 years, membership has expanded from three to over fifty institutions across twenty economies.

While the network has been instrumental in advancing HTA in Asia, its sustainability depends on voluntary contributions. This inclusive and voluntary model represents one of the network’s core strengths, promoting shared ownership and broad regional representation. However, it also poses challenges in ensuring long-term engagement and operational stability. HTAsiaLink must proactively address emerging challenges and strengthen its value to members to remain relevant in an evolving healthcare landscape.

This review reflects on the strategic planning process not merely as an internal milestone for the network, but as a case study with broader implications for the HTA community. The experience sheds light on how voluntary regional networks can engage members, align diverse priorities, and build consensus in the absence of formal governance or dedicated funding structures. These reflections may be useful for other networks facing similar circumstances and seeking ways to enhance collaboration and long-term planning. This commentary outlines the process of developing the network’s first strategic plan, focusing on how member input, contextual factors, and available evidence were used to shape the final product. It aims to contribute to ongoing conversations about how HTA networks can evolve their governance and strategies in ways that are responsive to member needs and adaptable to changing environments.

## Structured development process

**Figure 1.**
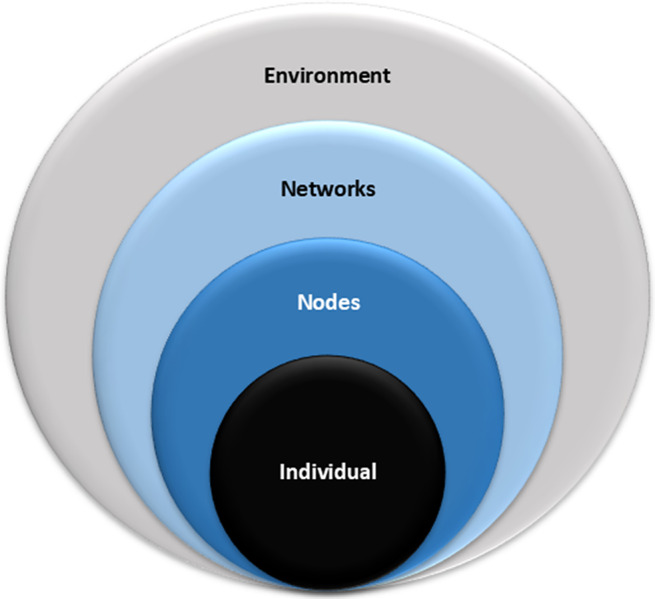
Elements of the Individual, Node, Networks, and Environment (INNE) framework used to design data collection tools and analyze data across four capacity building levels.

The strategic plan was developed through three key steps: (i) analyzing strategic frameworks of other HTA networks; (ii) assessing members’ needs in capacity building and HTA institutionalization; and (iii) formulating and collecting input for a 5-year roadmap based on these findings. The Individual, Node, Networks, and Environment (INNE) framework was adopted as an analytical tool to organize and interpret efforts aimed at capacity building across multiple levels (3). Recognizing that different levels require distinct strategies, the INNE framework (see Figure 1) was applied to both qualitative and quantitative methodologies. It guided the targeted review of strategic plans from nine international networks and organizations focused on HTA and health policy, identifying best practices in stakeholder engagement, capacity building, and policy alignment (refer to the *full report* (4)). In addition, it shaped the design of a survey and key informant interviews (KIIs) aimed at assessing member engagement levels, expectations, and concerns. This framework enables a focused exploration of network members’ perspectives and needs across multiple dimensions of capacity building.

HTAsiaLink’s strategic development can be seen in the context of broader movements in global HTA collaboration. To provide this context, we reviewed the missions and activities of organizations such as Health Technology Assessment international (HTAi) (5), a global society promoting HTA; the International Network of Agencies for Health Technology Assessment (INAHTA) (6), which connects government HTA agencies worldwide; the International Society for Pharmacoeconomics and Outcomes Research (ISPOR) (7), an organization advancing health economics and outcomes research; the Patient-Centered Outcomes Research Institute (PCORI) (8), which funds stakeholder-engaged health research; the International Decision Support Initiative (iDSI) (9), a partnership supporting HTA capacity in low- and middle-income countries; the United Nations Development Programme (UNDP) (10), a United Nations agency promoting sustainable development; and the Africa Centres for Disease Control and Prevention (Africa CDC) (11), a regional public health agency focused on strengthening disease control in Africa, all of which prioritize HTA in healthcare decision-making. While these initiatives vary in structure and geographic focus, they share a common goal of advancing HTA. Their diversity underscores the notion that there is no one-size-fits-all approach to achieving this objective.

HTAsiaLink embodies a unique model of collaboration that distinguishes it from other global HTA networks in its approach and operational principles. First, HTAsiaLink’s inclusive model broadens access to network resources and opportunities by minimizing barriers through the absence of membership and registration fees. Second, it brings together a diverse range of institutions, including HTA agencies, academic bodies such as universities, and public research centers, many of which operate independently of commercial interests. This structure cultivates a neutral and collaborative environment that encourages open dialogue and facilitates knowledge sharing. However, this model presents challenges for HTAsiaLink, especially concerning its long-term financial sustainability.

Engagement of members in shaping the network’s roadmap, facilitated through surveys and KIIs, led to the identification of five key themes: HTA capacity building; collaboration; knowledge exchange; HTA utilization; and network sustainability. The results further underscore the importance of building on existing efforts while simultaneously exploring targeted initiatives to address emerging needs. Moreover, they highlight the need to create opportunities for members to actively contribute to decision-making and take on leadership roles within the network, fostering a sense of ownership and long-term commitment.

These findings informed the network’s strategic goals and actionable plans. Continuous feedback loops were established, incorporating input from Board members and experts to ensure the plan remains relevant and adaptable. The final strategic plan is data-driven, responsive to member needs, and designed for periodic reviews and refinements.

## The way forward: Positioning HTAsiaLink in the growth of HTA in Asia

**Figure 2.**
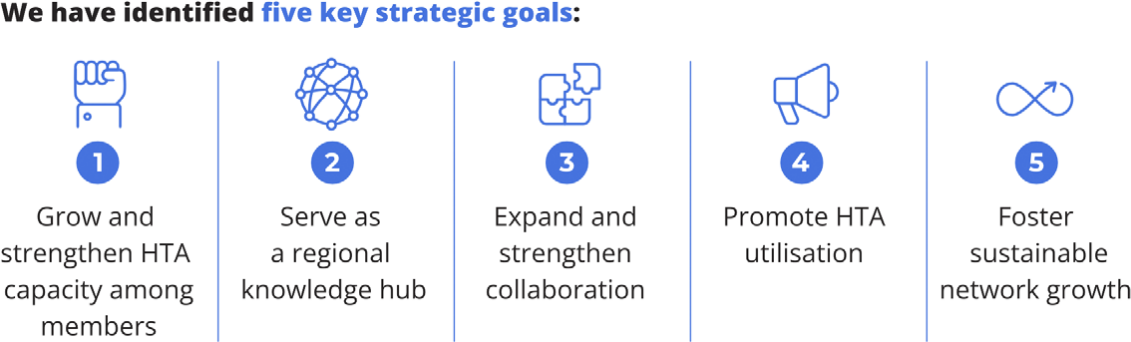
Five strategic goals outlining key objectives to guide the network’s direction over the next 5 years, ensuring alignment with the broader vision and mission to support growth and broaden impact.

The strategic plan provides a roadmap for HTAsiaLink’s growth and impact, outlining five strategic goals (see Figure 2) and four key actions with defined metrics for success (available in Annex 1 of the Supplementary Material) to be achieved by 2030. To support monitoring and accountability, both quantitative and qualitative indicators have been established. For example, member engagement will be tracked through the indicator “number of highly engaged members,” with verification based on an annual membership engagement survey assessing participation frequency and event satisfaction. The target is for members to participate in at least three network events or collaborative projects per year. This indicator contributes to the broader outcome of fostering an engaged and growing network characterized by a supportive environment, shared ownership, and transparency.

Achieving the strategic goals requires active participation from members, guided by the Board, Secretariat, and Editorial Team. The Board, consisting of nine members from nine economies, provides strategic leadership, decision-making support, and oversight to ensure alignment with HTAsiaLink’s mission, and is elected after each conference in accordance with the by-laws. The Secretariat and Editorial Team, both based at HITAP, manage daily operations, communications, coordination, and knowledge dissemination.

Building on its strengths, the network will enhance capacity building and knowledge exchange while exploring new approaches tailored to diverse member needs. Active engagement will be key to sustaining these efforts, with initiatives such as topic nominations to integrate member input into network activities, volunteering to take on leadership roles to enhance inclusivity through event coordination, and mentorship programs to expand learning opportunities for early-career researchers. These initiatives will be instrumental in cultivating the next generation of network leaders and ensuring the network’s long-term sustainability.

HTAsiaLink will continuously strengthen collaborations with non-member stakeholders, for example, policymakers, academics, other non-profit international organizations, and like-minded networks. This effort aims at expanding the network’s influence, fostering broader knowledge exchange, and promoting cross-disciplinary partnerships. A monitoring and evaluation framework has been developed to track progress, categorizing actions into easy wins, mid-priority, and long-term objectives. This structured approach will enable timely adjustments and continuous improvement, ensuring the network remains a key driver of HTA advancement in the region.

A review of various strategic plans reveals common priorities, including strengthening HTA, improving health outcomes, advancing research, aligning policies, and fostering global partnerships. Organizations employ different strategies to achieve these goals, such as inclusive and participatory planning, active stakeholder engagement, and strong data-driven approaches. By drawing on these insights, HTAsiaLink can refine its strategic direction, ensuring it remains comprehensive, forward-thinking, and aligned with international best practices.

Achieving international standards in HTA requires HTAsiaLink to continuously elevate its practices, adopt global best practices, and align its activities with international benchmarks. By fostering a dynamic network, HTAsiaLink can navigate challenges, seize emerging opportunities, and solidify its role as a leading platform for advancing HTA in the region. While HTAsiaLink’s strategic plan is ambitious, it is built on a foundation of collaboration, evidence-informed policymaking, and capacity building. Compared to other strategic plans, HTAsiaLink’s approach is uniquely positioned to address regional needs while incorporating best practices globally. Its adaptability allows for application in other HTA networks, offering valuable lessons for policy alignment in diverse healthcare environments. Despite challenges such as limited technical capacity and the need for continuous stakeholder engagement, there are significant opportunities for HTAsiaLink to drive meaningful progress. In the current global geopolitical landscape, where healthcare demands continue to rise due to population aging, technological advancements, and increasing expectations, ensuring the efficient and sustainable use of healthcare budgets is crucial. HTAsiaLink plays a vital role in addressing these challenges by fostering regional collaboration, strengthening institutional capacity, and promoting knowledge exchange, ensuring that HTA remains responsive to the evolving healthcare landscape and policy needs. With a well-defined strategic plan, HTAsiaLink is positioned to enhance HTA implementation efficiently, ensuring that healthcare decisions remain informed, equitable, and sustainable across the Asia-Pacific region.

## Supporting information

Sitanggang et al. supplementary material Sitanggang et al. supplementary material

## Supplementary material

The supplementary material for this article can be found at http://doi.org/10.1017/S0266462325100573.
