# Supplementary material for: Charting the way forward for HTA in Asia-Pacific: HTAsiaLink’s strategic plan
Source: Int J Technol Assess Health Care. 2025 Dec 19;41(1):e87. doi: 10.1017/S0266462325100573 (PMC12723306; doi:10.1017/S0266462325100573)
Supplement: Sitanggang et al. supplementary material [file S0266462325100573sup001.docx]

**Annex**

Annex 1: Impact metrics for measuring key actions and activities, detailing the responsible teams, their alignment with strategic goals, the timeline over a 5-year period, and expected outputs and outcomes.

| **Activity** | **Indicator** | **Responsible team** | **Means of verification** | **Baseline** | **Output/Target** | **Strategic Goals** | | | | | **Timeline (Year)** | | | | | **Outcome** |
| --- | --- | --- | --- | --- | --- | --- | --- | --- | --- | --- | --- | --- | --- | --- | --- | --- |
|  |  |  |  |  |  | 1 | 2 | 3 | 4 | 5 | 2025 - 2026 | 2026 - 2027 | 2027 - 2028 | 2028- 2029 | 2029 - 2030 |  |
| **1. Strengthen and Advance Capacity Development Initiatives** | | | | | | | | | | | | | | | | |
| 1.1 Maintain Core Activities: Sustain flagship programs yearly such as the annual HTAsiaLink Conference, workshops, and webinars | 1) Number of HTAsiaLink-hosted events, training programs, and programmatic activities | Secretariat | Review official calendars and schedules for events and activities maintained by Secretariat. | HTAsiaLink hosted events from the most recent events (e.g., conference attendance in 2023) | 1 conference, at least 1 training program, and at least 1 webinar per year | 🗸 | 🗸 |  |  |  | 🗸 | 🗸 | 🗸 | 🗸 | 🗸 | Increased engagement, particularly among new members and those from underrepresented regions |
| 1.2 Conduct Regular Landscape Analyses of HTA Needs: Identify and address knowledge gaps of members by conducting survey analysis as needed | 1) Percentage of member organizations responding to the survey | Secretariat | Survey reports and data summaries, as needed (at least twice in a 5-year period) | Not applicable | At least 1 member from each country to respond to the survey |  | 🗸 | 🗸 |  | 🗸 | 🗸 |  | 🗸 |  | 🗸 | Improved understanding of regional HTA priorities and challenges among members |
|  | 2) Needs and priorities identified for the relevant time frame | Secretariat | Survey reports and data summaries, as needed (at least twice in a 5-year period) | Not applicable | Report summarizing HTA needs and knowledge gaps |  | 🗸 | 🗸 |  | 🗸 | 🗸 |  | 🗸 |  | 🗸 | Improved understanding of regional HTA priorities and challenges among members |
| 1.3 Broaden Learning Opportunities: Introduce and implement innovative formats such as modular courses, structured mentorship and internships | 1) Number of new HTAsiaLink hosted events outside of core activities | Secretariat and organizing team | Review official calendars and schedules for events and activities maintained by Secretariat | Existing formats primarily include early career researcher program (ECR) | At least 1 additional activity/event per year | 🗸 | 🗸 |  | 🗸 |  |  | 🗸 | 🗸 | 🗸 | 🗸 | Improved HTA competencies among members, particularly early-career professionals and those in emerging HTA settings |
| **2. Improve Communication and Knowledge Exchange** | | | | | | | | | | | | | | | | |
| 2.1 Develop Engaging Communication Strategies: Develop and maintain accessible digital platforms that centralize resources, disseminate information, and enable members to contribute actively to the network’s activities; launch targeted content for further engagement; Showcase outputs and outcomes of network activities | 1) HTAsiaLink website with engagement features for members | Secretariat and Editorial team | Record of engagement features (website screenshots) | Current version of HTAsiaLink website as of December 2024 | Completion of Phase II website for HTAsiaLink members, with an engagement feature |  | 🗸 | 🗸 | 🗸 |  | 🗸 |  |  |  |  | Members are able to stay up to date and receive information more efficiently and maintain professional connection among members |
| 2.2 Expand Knowledge Sharing Platforms: Establish a registry by 2025 to aggregate upcoming, ongoing, and completed HTA studies conducted by members as a region-wide knowledge sharing platform | 1) Completion of the registry | Secretariat, registry development team (IT team) | Functional registry with real-time access to aggregated HTA studies | Current status of knowledge sharing within the network (e.g., informal data exchanges, limited access to consolidated resources) | A fully operational and accessible HTA registry by 2025 |  | 🗸 | 🗸 | 🗸 |  | 🗸 |  |  |  |  | - Improved regional collaboration through easier access to HTA research.  - Reduction in duplicated research efforts by identifying existing studies |
|  | 2) Number of HTA studies aggregated in the registry annually | Secretariat, registry development team (IT team) | A list of HTA studies in the registry | Metrics of first year of operation | Comprehensive dataset of member- contributed HTA studies |  | 🗸 | 🗸 | 🗸 |  | 🗸 | 🗸 | 🗸 | 🗸 | 🗸 | - Improved regional collaboration through easier access to HTA research.  - Reduction in duplicated research efforts by identifying existing studies |
|  | 3) User usage | Secretariat, registry development team (IT team) | Metrics (e.g., number of logins, searches, and data downloads) | Metrics of first year of operation | At least 5 additional new users per year |  | 🗸 | 🗸 | 🗸 |  | 🗸 | 🗸 | 🗸 | 🗸 | 🗸 | - Improved regional collaboration through easier access to HTA research.  - Reduction in duplicated research efforts by identifying existing studies |
|  | 4) User experience through survey | Secretariat | Feedback from members through surveys on registry usability and relevance after launch of registry | Metrics of first year of operation | At least 60% of respondents report registry-use to be accessible, satisfactory, and applicable |  | 🗸 | 🗸 | 🗸 |  | 🗸 |  | 🗸 |  | 🗸 | - Improved regional collaboration through easier access to HTA research.  - Reduction in duplicated research efforts by identifying existing studies |
| 2.3 Explore and Strengthen Partnerships: Strengthen current and explore new partnerships through formal collaborations such as Memoranda of Understanding (MoU) | 1) Number of joint activities or projects initiated as a result of partnerships | Secretariat | Reports or documentation on joint activities and collaborative projects | 2 joint activities with external partners annually as of 2024 (HTAi-HTAsiaLink webinar, guideline development) | At least 2 joint activities with external partners per year |  | 🗸 | 🗸 | 🗸 |  | 🗸 | 🗸 | 🗸 | 🗸 | 🗸 | Strengthened regional and international collaboration to support HTA growth |
| **3. Expand and Sustain the Network** | | | | | | | | | | | | | | | | |
| 3.1 Expand and Maintain Membership and Regional Representation: Implement targeted recruitment strategies to attract to all professional levels from emerging HTA contexts, focusing on underrepresented countries and nascent HTA organizations. Provide access to network resources, expertise, and mentorship to drive HTA growth across the region. Raise awareness of membership benefits and ensure a seamless onboarding experience for new members. Ensure inclusiveness and strengthen regional representation | 1) Annual increase in organizational and associate memberships | Secretariat | Record and review list of memberships | 50 organizational and associate members | At least 2 organizational and/or associate members annually | 🗸 |  |  |  | 🗸 | 🗸 | 🗸 | 🗸 | 🗸 | 🗸 | Engaged and growing network with supportive environment, shared ownership, and transparency. |
|  | 2) Number of highly engaged members | Secretariat | Conduct annual membership engagement survey to assess participation frequency and event satisfaction | Metrics of first year of operation | Number of members participating in at least 3 network events/collaborative projects per year |  |  | 🗸 |  | 🗸 | 🗸 | 🗸 | 🗸 | 🗸 | 🗸 | Engaged and growing network with supportive environment, shared ownership, and transparency. |
| 3.2 Develop Standardized Reporting and Guidelines: Establish clear and consistent reporting standards and guidelines for network activities to ensure consistent quality and efficiency and ensure documentation of institutional memory | 1) Number of guidelines for hosting network activities | Secretariat | Review existing activity materials on the process for hosting network events such as annual conference and webinar | Existing documentation on scoring abstract at conference, Terms of Reference (ToR), and conference proceedings | 1 guideline for hosting Annual Conference 1 logo guideline 1 conference summary/proceeding template |  |  |  |  | 🗸 | 🗸 | 🗸 | 🗸 | 🗸 | 🗸 | Standardized reporting and guidelines for network activities' documentation, which can be easily accessible without the need to re-develop and maintain consistency |
| 3.3 Empower Leadership: Cultivate a sense of inclusivity and professional belonging by providing members with opportunities to contribute to decision-making and leadership within the network. Encourage volunteer participation to facilitate a wider range of network events and activities beyond the annual conference | 1) Number of activity proposals led by members | Network members with the support by Secretariat | Annual topic nomination and volunteer survey; Record and track a list of annual activities | Metrics of first year of operation | At least 1 member-led activity per year |  |  | 🗸 | 🗸 |  | 🗸 | 🗸 | 🗸 | 🗸 | 🗸 | Members gain leadership skills to develop initiatives to enhance network and respective organizational capacity in driving HTA-related practices |
| 3.4 Foster Leadership Development: To identify and cultivate future leaders among members to support emerging leaders and foster a culture of volunteerism and inclusivity | 1) List of representatives/leaders who will engage with HTAsiaLink for the duration of the strategic plan | Secretariat with the Board's guidance | Actively reach out to representatives to identify potential members for leadership in the next 3-5 years | Metrics of first year of operation | At least 2 people per country who can represent and engage with HTAsiaLink |  |  |  |  | 🗸 | 🗸 | 🗸 | 🗸 | 🗸 | 🗸 | Cultivating future leaders among members to enhance and sustain the network's representation and impact at the regional level and beyond |
| 3.5 Promote Member Ownership and Inclusivity: Empower network members and foster a sense of ownership and engagement through their involvement in the network’s activities | 1) Number of members participating in providing feedback and inputs upon request | Secretariat | Record and track the number and frequency of members participating in feedback process | Metrics of first year of operation | At least 40% response rate from members per activity |  |  | 🗸 |  | 🗸 |  | 🗸 | 🗸 | 🗸 | 🗸 | Strong partnerships and accountability among members in driving diverse network’s initiatives |
| 3.6 Diversify Funding Sources: Support key initiatives and members by seeking grants from global and international public organizations through joint projects and partnerships. | 1) Number of joint research activities | Project focal point with the support by Secretariat | Record and track a list of joint research projects | Metrics of first year of operation | At least 3 joint research projects with fundings for organizations involved by 2030 | 🗸 |  | 🗸 |  |  |  |  |  |  | 🗸 | Increased availability of resources to conduct research relevant to regional needs and sustained collaboration |
| 3.7 Explore Financial Models for the Network Activities: Identify financial management systems to support activities for transparency and effective resource allocation, with regular evaluations and adjustments to optimize funding strategies and secure long-term financial stability if the funding is managed by the network | 1) Network's financial management system once funding is received | Party appointed by the Board | Board meeting minutes | Not available (currently no funds are managed at the network level) | A financial management system |  |  |  |  | 🗸 |  |  |  |  | 🗸 | Central system to manage funding to achieve financial sustainability and transparency |
| **4. Increase HTA Impact** | | | | | | | | | | | | | | | | |
| 4.1 Monitor and Evaluate the Network’s Impact: Maintain records of activities, outputs (e.g. guidelines, publications, reports, etc.), and outcomes (e.g. level of engagement, the use of outputs, etc.) of the network, and conduct evaluations as deemed necessary. | 1) Number of peer-reviewed publications of projects conducted under the auspices of HTAsiaLink | Secretariat and project focal points | Record and search for published papers in the registry | Metrics of first year of operation | At least 3 peer-review publications by 2030 |  |  | 🗸 | 🗸 |  |  | 🗸 | 🗸 | 🗸 | 🗸 | Improved the ability to track engagement of the network |
|  | 2) Number of users of HTAsiaLink products - newsletter | Secretariat | Record number of views of the Newsletter | Metrics of first year of operation | At least 50 views per Newsletter | 🗸 |  |  | 🗸 |  | 🗸 | 🗸 | 🗸 | 🗸 | 🗸 | Improved the ability to track engagement of the network |
|  | 3) Traffic metrics of HTAsiaLink website | Editorial team | Track the number of views on materials on the network website | Metrics of first year of operation | At least 50 views on each material on the website per month | 🗸 | 🗸 |  | 🗸 |  | 🗸 | 🗸 | 🗸 | 🗸 | 🗸 | Improved the ability to track engagement of the network |
|  | 4) Develop impact stories to showcase impact of network | Secretariat and Board members | Review activities/collect data | Not available (currently not being conducted) | At least one impact story |  |  |  |  |  |  |  | 🗸 |  |  | Improved the ability to track engagement of the network |
| 4.2 Increase External Engagement: Organize platforms and activities to engage with evidence users, such as decision makers, and other non-member stakeholders to ensure alignment with their interests. Coordinate targeted pre-conference sessions and training, as well as share resources and collaborate through digital platforms. | 1) Number of policymakers participating in HTAsiaLink events | Event organizers and Secretariat | Record participants list | First year of operation | At least 5 policymakers per event |  | 🗸 |  | 🗸 |  | 🗸 | 🗸 | 🗸 | 🗸 | 🗸 | Increased awareness of HTA utilization in health policy and foster future collaborations with stakeholders |
|  | 2) Number of events that involve non-member participants | Event organizers and Secretariat | Review official calendars and schedules maintained by secretariat | First year of operation | At least 1 event involved non-member participants per year | 🗸 | 🗸 | 🗸 | 🗸 |  |  | 🗸 | 🗸 | 🗸 | 🗸 | Increased awareness of HTA utilization in health policy and foster future collaborations with stakeholders |
| 4.3 Increase Visibility Outside the Network: Increase HTAsiaLink's visibility and impact by actively participating in external activities and events to engage with broader health policy and HTA communities. | 1) Number of HTAsiaLink representatives engaged in external activities/events | HTAsiaLink representatives | Track and report on the participated events | 1 activity (RedETSA 2024) | At least 2 external events participated by HTAsiaLink representatives per year |  |  | 🗸 | 🗸 |  |  | 🗸 | 🗸 | 🗸 | 🗸 | Increased awareness of HTA utilization in health policy and foster future collaborations with stakeholders |
